# Erie County Medical Society

**Published:** 1869-01

**Authors:** 


### Erie County Medical Society.

At the Annual Meeting of the Erie County Medical Society, held on Tuesday, 12th inst., the following officers were elected for the ensuing year:

President, O. K. Parker; Vice President, J. F. Miner; Secretary, M. G. Potter; Treasurer, Wm. Ring; Librarian, J. B. Samo.

Primary Board—E. R. Barnes, H. R. Hopkins, W. C. Phelps.

Censors—Anatomy, Physiology and Surgery, Sandford Eastman; Practice of Medicine and Obstetrics, John Boardman; Materia Medica and Botany, M. O. Potter; Medical Jurisprudence, J. Smith; Chemistry and Pharmacy, J. R. Lothrop.

A paper on Pyromania was read by Dr. W. C. Phelps. Dr. J. S. Trowbridge read a memoir of his father, the late Dr. Josiah Trowbridge, which was ordered to be published in pamphlet form.

The following gentlemen were elected to membership on compliance with the By-Laws: Hiram Tabor, G. H. Gail, Jacob Worp Van Peyma, Elias T. Dorland, Albert S. Rogers, Wm. Osborn Taylor, Henry B. Murray and W. S. Talbott.

Valedictory Address by the President, Dr. John Boardman.

Committee to choose Orator for the Semi-Annual Meeting reported names of Dr. H. R. Hopkins for orator, and Dr. Davis E. Chace for substitute.

