# Correction of Name

**Published:** 1869-02

**Authors:** 


CORRECTION OF NAME.—In our publication of the names of the physicians who joined the Erie County Medical Society at its last meeting, the name of Dr. Wm. H. Gale of Aurora, was printed G. H. Gail.

